# A Man Who Is Fed through a Funnel

**Published:** 1893-04

**Authors:** 


A MAN WHO IS FED THROUGH A FUNNEL.—The case of Mr. John Tainsh, of Virginia, who has recently undergone an unusual and successful surgical operation at the Maryland University Hospital is being watched with great interest by the medical profession, and a number of physicians from other cities have come to Baltimore to examine it. Several months ago Mr. Tainsh became unable to swallow food, and for a time he was kept alive on milk, which was poured through a slender tube inserted into his throat. When brought to the hospital but little hope was at first entertained for his recovery. Dr. Louis McL. Tiffany diagnosed the case and found that the trouble was caused by the æsophagus having become choked. An operation upon the æsophagus was considered dangerous on account of the proximity of the heart, and Dr. Tiffany concluded the only way to save Mr. Tainsh from starvation was to make a new entrance to the stomach for the food.

This he did by cutting the abdomen and inserting a silver tube into the stomach. Through this tube Mr. Tainsh now receives nourishment, and he is growing strong. When he is hungry he stretches himself at full length upon his back and the physician in attendance puts a funnel to the tube and pours in milk, soup and other food. Mr. Tainsh's hunger is at once appeased by this method. He walks out every fine day.

